# Studying the microbiome of suppressive soils against vascular wilt, caused by *Fusarium oxysporum* in cape gooseberry (*Physalis peruviana*)

**DOI:** 10.1111/1758-2229.13195

**Published:** 2023-09-07

**Authors:** Daniel Bautista, Diana García, Lorena Dávila, Alejandro Caro‐Quintero, Alba Marina Cotes, Adriana González, A. Paola Zuluaga

**Affiliations:** ^1^ Corporación Colombiana de Investigación Agropecuaria, Agrosavia, Centro de Investigación Tibaitatá Bogotá Colombia; ^2^ Department of Biological Sciences Universidad de Los Andes Bogotá Colombia; ^3^ Department of Biology Universidad Nacional de Colombia Bogotá Colombia

## Abstract

Cape gooseberry (*Physalis peruviana*) is Colombia's second most exported fruit, with a market worth 37.8 million USD in 2021. *Fusarium oxysporum* f sp. *physalis* (*Foph*) is arguably the most devastating pathogen causing losses of up to 80%. Managing this disease is challenging due to pathogen resistance or the reduced efficacy of commercial fungicides and the production of resistant structures allowing pathogen survival in the soil for up to 30 years. Thus, new methods of control are necessary. Two cape gooseberry farms (organic vs. conventional) were detected free from *Foph* in Nariño. We hypothesize that the soil microbiome might have a suppressive effect against vascular wilt, caused by *Foph*. To test this, farm soils were propagated by adding 10% farm soil and 90% peat soil. Then, peat soil (control) and propagated soils were inoculated with *Foph*. A decrease of 65%–68% in disease incidence and a 70% in disease severity reduction was observed in seedlings grown in propagated soils compared to peat soil. We then used next‐generation sequencing to study the soil microbiome to understand the possible mechanisms for disease suppression of propagated soils. We conclude that despite the high diversity of soil microbiomes, the relative abundance of some taxa might be a more important indicator of disease suppression than the presence of specific taxa.

## Graphical abstract

The soil microbiome contributes to plant growth, disease suppression and in general, to the ecosystem health. To understand the possible mechanisms for vascular wilt suppression, we studied the soil microbiome of two contrasting soils: conducive versus suppressive for *Fusarium oxysporum* f. sp *physalis*. We conclude that despite the high diversity of soil microbiomes, the relative abundance of some taxa might be a more important indicator of disease suppression than the presence of specific taxa.

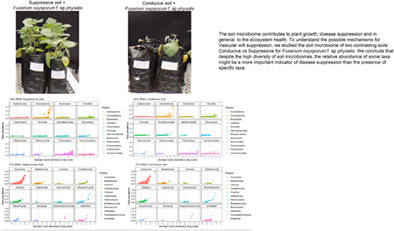

## INTRODUCTION

The soil microbiome contributes to plant growth, bioremediation, disease suppression and in general, to the ecosystem health (Naylor et al., 2020). Thus, plants rely on soil microbiota for specific functions and traits, such as disease suppression, and in exchange, plants exudate up to 21% of their photosynthetically fixed carbon to the rhizosphere (Mendes et al., 2011). The growth of plant pathogens in soil can be reduced through competitive interactions with the soil microbial community, resulting in suppressive soils (Döring et al., 2020; Naylor et al., 2020; Siegel‐Hertz et al., 2018). Biological suppressive soils are those in which, because of their microbial composition, a pathogen does not establish, or establishes but does not persist, or persists causing a minor disease, or causes disease, but the disease declines with successive cropping of a susceptible host despite the presence of the pathogen (Baker & Cook, 1974). There are two types of suppressive soils, specific and general. Specific suppression results from either individual species or a select group of microorganisms and is transferable by adding small amounts of suppressive soil, 1% or 10%, to conducive soil. Transferability is the main difference between general and specific suppressive soils, and that suppression is due to a population rather than a community (Cook, 2014). One of the best‐studied examples of typical suppressive soils is a take‐all disease in wheat, where *Pseudomonas* spp., bacteria secrete a natural phenol 2,4‐diacetylphloroglucinol, which suppresses the fungal pathogen *Gaeumannomyces graminis* var. *tritici* (Palma‐Guerrero et al., 2021). For decades, the region of Châteaurenard, in France has been studied for its suppressiveness to *F. oxysporum* (Alabouvette, 1986). The suppressive effect was attributed to the presence of non‐pathogenic *F. oxysporum* exerting trophic competition and fluorescent *Pseudomonas‐producing* metabolites such as pyoverdins, antifungal phenazines and iron‐chelating siderophores, making iron access difficult for the pathogenic *F. oxysporum* (Alabouvette, 1999; Lemanceau & Alabouvette, 1993; Mazurier et al., 2009).

On the other hand, general suppression is due to the collective competitive and antagonistic activity of the whole soil microbiome, competing with the pathogen (Schlatter et al., 2017). It is not transferable in small amounts of soil, and it is generally effective against a broad range of pathogens. General suppression is based on multitrophic interactions, but the microbes and mechanisms involved in disease suppression are unknown for most general‐suppressive soils. Until the 2010s, most of the studies were focused on individual beneficial microorganisms, because of the lack of tools to study the whole soil microbiome (Toyota & Shirai, 2018). However, the advent of new tools, such as next‐generation sequencing, has allowed the study of the entire rhizosphere microbiome, widening our understanding of the complex mechanisms governing suppressive soils. One of the main findings was that soil suppressiveness is not dependent on a single bacterial taxon or group and is most likely controlled by microbial consortia (Mendes et al., 2011). This is supported by the observation that bacterial strains, lacking activity against pathogens when tested alone, can act synergistically when part of microbial consortia (Mendes et al., 2011). Another finding was the identification of fungi and bacterial genera associated with suppressive soils. For instance, a couple of comparative studies between suppressive and conducive (non‐suppressive) soil microbiomes against Fusarium wilt found several of the fungal and bacterial genera detected exclusively or more abundantly in the Fusarium wilt‐suppressive soil included genera known for their activity against *F. oxysporum* (Siegel‐Hertz et al., 2018; Xiong et al., 2017).

Cape gooseberry (*Physalis peruviana*) belongs to the Solanaceae family and is originally from Peru. It is well adapted to Colombia, where it is grown in five states: Antioquia, Boyacá, Cundinamarca, Nariño and Santander from 1500 to 3000 m above sea level, and it has become the second most exported fruit after bananas, with a market worth close to 37.8 million USD annually (Arias et al., 2015; Fructidor, 2022). However, vascular wilt caused by the fungal pathogen *Fusarium oxysporum* f sp. *physalis* (*Foph*) is arguably the most devastating pathogen for cape gooseberry, causing up to 80% of loss of the crop (Diaz et al., 2012; Osorio‐Guarín et al., 2016; Simbaqueba et al., 2018). Managing this disease is very challenging due to pathogen resistance to commercial fungicides, the low efficacy of commercially available fungicides and the production of resistant structures called chlamydospores that allow the pathogen to survive on the soil for extended periods (McGovern, 2015). This has resulted in producers moving their crops to new regions to avoid the pathogen, with two detrimental consequences: spreading the disease to new areas and abandoning farming land (Simbaqueba et al., 2018). Despite this, Nariño has been the only department where this pathogen has not been reported. Thus, we hypothesize that Nariño might have specific suppressive soils against *Foph*. To test this hypothesis, we used next‐generation sequencing of phylogenetic markers to study the soil microbiome from two farms with contrasting managing strategies: organic versus conventional in Nariño state, where *Foph* incidence has not been reported.

## EXPERIMENTAL PROCEDURES

### Plant material

Commercial cape gooseberry (*P. peruviana*) seeds ecotype Colombia was used. Seeds were surface sterilized with 70% ethanol for 2 min, followed by a 20‐min wash in 3% Sodium hypochlorite and three washes with sterile distilled water. Seeds were sown in seedling trays with pure peat. A foliar fertilizer at a concentration of 0.5 mL per litre of water was applied weekly, starting in 1‐month‐old plants. Three‐week‐old seedlings were transplanted into bags containing either suppressive or conducive soils for experiments.

The experiments were done in a greenhouse of the Corporación Colombiana de Investigación Agropecuaria Agrosavia, Mosquera (4°41′45″ N 74°12′12″ W). With a natural photoperiod of 12 h (photosynthetically active radiation 1500 μm—1 s—2 at noon), and an average temperature of 15°C at night and 25°C during the day with relative humidity of ~72%.

### Inoculum preparation and soil inoculation

*F. oxysporum* f sp. *physalis* Map 5 (*Foph*; Simbaqueba et al., 2018) was cultured in Potato dextrose agar (PDA Difco®) at 25°C for 8 days. Then, three plugs of 0.5 cm were added to 300 mL of potato‐dextrose broth (PDB Difco®) in a 1‐L Erlenmeyer and kept at 25°C for 1 week in agitation at 125 rpm. The inoculum was filtered using Whatman filter paper, the microconidia were measured in a Neubauer chamber, and the concentration was adjusted to 1 × 10^6^ microconidia mL^−1^. Next, 900 g of soil was inoculated with 100 mL of the spore suspension.

### Suppressive soil collection

Rhizospheric soil was collected from two gape gooseberry farms in Nariño, Colombia, with the required collection permit 1466 from 2014 given by ANLA to Agrosavia. The two farms had contrasting management strategies. One farm had organic management (certified since 2008 by ICA) and was in Gualmatán (Nariño), and the other farm had conventional management located in Puerres (Nariño). These zones have a historical absence of Fusarium wilt and have been pointed out as possible sources for suppressive soils. Rhizospheric soil from conventional farms was sampled from 12‐month‐old healthy cape gooseberry plants by unearthing the plants, removing the unattached soil surrounding the plant and collecting only the soil that was firmly attached to the root system (Timonin, 1946). Rhizospheric soil was sampled from 5‐month‐old healthy cape gooseberry plants from the organic farm by unearthing the plants, removing the unattached soil surrounding the plant and collecting only the soil that was firmly attached to the root system. Soils were kept at 4°C during transportation and storage.

### Suppressive soil propagation (propagated soil)

Because we wanted to test whether Nariño soils presented specific suppression against *Foph*, we mixed 10% of the rhizospheric soil collected from organic farms with 90% of commercially available soil (conducive soil). The mix was then amended with rice husk in a 3:1 proportion. The addition of rice husk is important to allow optimal oxygenation of soil for pathogen growth (Moreno‐Velandia et al., 2019). We call this mix propagated soil used to test our hypothesis for specific soil suppression. Soils were kept at 4°C until used.

### Soil physicochemical analyses

Three samples were taken at each farm to determine the physicochemical properties of both farm soils at a depth of 10 cm. Soil samples were kept at 4°C during transportation and storage until they were analysed at the water and soil laboratory in Agrosavia (Corporación Colombiana de Investigación Agropecuaria). Texture, organic matter content, pH, Ca, Mg, K, Na, Fe, B, Zn, S, P and cation‐exchange capacity were measured. Additionally, the physicochemical properties of the propagated soil mix from both farms used for these experiments and the conducive soil (commercial soil) was also analysed (Table 1).

**TABLE 1:** Physicochemical soil analyses for the soils used in this study.

Properties	Suppressive soils		Propagated soil (10% suppressive soil + 90% conducive soil)		Conducive soil
	(Conventional farm)	(Organic farm)	(Conventional farm)	(Organic farm)	
Texture	Clay loam soil		Silty clay loam soil		Silty clay loam soil
CEC^a^ (mmol/g)	10.11	14.61	8.08	8.27	6.82
Organic matter (%)	3.37	2.82	10.18	10.13	9.86
pH	4.89	6.07	5.42	5.42	5.24
Ca_2_ (mmol/g)	6.06	10.21	3.78	3.88	3.67
Mg_2_ (mmol/g)	1.64	1.96	2.3	2.37	1.68
K (mmol/g)	1.63	2.16	0.67	0.6	0.8
Na (mmol/g)	0.1	0.28	0.76	0.88	0.26
Fe (mg/kg)	314.56	215.35	425.01	426.11	402.42
B (mg/kg)	0.47	0.84	0.26	0.26	0.07
S (mg/kg)	6.41	19.13	67.31	74.52	62.63
P (mg/kg)	311.26	71.44	7.11	3.87	25.21
Zn (mg/kg)	3.93	6.88	9.19	10.86	6.13

^a^ Cation‐exchange capacity.

### Experimental design

Three‐week‐old cape gooseberry plants were transplanted to plastic bags containing either propagated soils (organic or conventional farms, see above) or conducive soil (commercial soil), inoculated and non‐inoculated with *Foph* Map5, in a randomized complete block design with four blocks (technical replicates). The whole experiment was repeated twice for a total of three biological repetitions. We had a total of six treatments: (1) propagated soil from organic, (2) propagated soil from organic inoculated with *Foph* Map5, (3) propagated soil from conventional, (4) propagated soil from conventional inoculated with *Foph* Map5, (5) conducive soil and (6) conducive soil inoculated with *Foph* Map5. Disease incidence was evaluated weekly for 79 days, and disease severity was calculated using a disease scale from 0 = no symptoms to 5 = plant wilting, chlorosis, and death (Moreno‐Velandia et al., 2019). For the microbiome studies, rhizospheric soil samples were taken from each treatment at the end of the trial (after 79 days). The area under the disease progress curve (AUDPC) was calculated from 15 to 79 days after inoculation as follows (Pedroza & Samaniego, 2009).

$$\text{AUDPC}=\sum_{i}\frac{Y_{i}+Y_{i+1}}{2}*\left(t_{i+1}-t_{i}\right),$$

where *Y*—disease index value; *t*—evaluation day.

AUDPC data from the three replicated experiments were analysed by Linear Model (GLM). The differences between the means were calculated by LSD Fisher's test with Bonferroni correction.

### Microbial genomic DNA isolation and library construction

The microbial genomic DNA was isolated using the DNeasy PowerSoil Kit from Qiagen following the manufacturers' instructions without modifications. A total of 0.25 g of soil per sample was used. DNA quality control was checked on a 1% agarose gel and nanodrop, ensuring that the 260/280 ratio was >1.8 and >2 for the 260/230 ratio.

Once the DNA quality was optimal for all samples, library construction to identify prokaryotes (bacteria and archaea) was done using the polymerase chain reaction (PCR) primers (F515/R806) developed to target the V4 region of the 16S SSU rRNA, which were determined to yield optimal community clustering (Pedroza & Samaniego, 2009). Primer 515F Forward: 5′‐GTGCCAGCMGCCGCGGTAA‐3′ and 806R Reverse: 5′‐GGACTACHVGGGTWTCTAAT‐3′ (Caporaso et al., 2012). To identify fungi (ascomycetes and basidiomycetes), the ITS3_KY02 5′‐GATGAAGAACGYAGYRAA‐3′ Forward primer and the ITS4_KY03 5′‐CTBTTVCCKCTTCACTCG‐3′ Reverse primer were used (Toju et al., 2012). For PCR amplification, Platinum™ *Taq* DNA Polymerase High Fidelity kit from Thermo Fisher Scientific was used. Each sample was amplified in triplicate. Conditions for 16S rRNA amplification were: Buffer 1×, MgSO_4_ 1.5 mM, dNTPs 0.2 mM, Reverse primer 0.2 mM, Forward primer 0.2 mM, Platinum Taq Hi‐Fi 2U and DNA 2 μL in a final volume of 25 μL. Amplification conditions were: Denaturalization at 94°C for 3 min; followed by 35 cycles of 94°C for 45 s, 50°C for 60 s, 72°C for 90 s and a final extension of 10 min at 72°C. Conditions for ITS amplification were: Buffer 1×, MgSO_4_ 3 mM, dNTPs 0.2 mM, Reverse primer 0.2 mM, Forward primer 0.2 mM, Platinum Taq Hi‐Fi 2 U and DNA 2 μL in a final volume of 25 μL. Amplification conditions were: Denaturalization at 95°C for 2 min; followed by 35 cycles of 95°C for 30 s, 55°C for 30 s, 72°C for 60 s and a final extension of 5 min at 72°C. To verify PCR amplification, PCR products were run in a 1.5% agarose gel and then cleaned up with magnetic beads before proceeding to the second PCR to add the barcodes to differentiate each sample during sequencing and data analyses. PCR conditions for both 16S rRNA and ITS: Buffer 1×, MgSO_4_ 1.5 mM, dNTPs 0.2 mM, Reverse primer 0.2 mM, Forward primer 0.2 mM, Platinum Taq Hi‐Fi 2U and DNA 5 μL in a final volume of 25 μL. Amplification conditions were denaturalization at 94°C for 3 min; followed by 12 cycles of 94°C for 45 s, 50°C for 60 s, 72°C for 90 s and a final extension of 10 min at 72°C. PCR products were run in a 1.5% agarose gel to verify PCR amplification and then cleaned up with magnetic beads. DNA quality and concentration were determined using Qubit fluorometric quantification. Libraries were combined at an equal concentration and paired‐end sequenced 2 × 250 nt Kit V2 with MiSEQ (Illumina) (Universidad del Bosque).

### Bioinformatic data analyses using the QIIME pipeline

Paired‐end sequences obtained on the Illumina MiSeq platform were analysed with QIIME 2‐2021.4 (Caporaso et al., 2010). First, forward and reverse reads were merged using BBMerge (using the options qtrim = r trimq = 8) (Bushnell, 2014), employing the adapter sequences for increased accuracy and soft quality trimming at the right end of the reads for better merging. Then, reads from both 16S rRNA and ITS sets were imported independently using the *demux* plugin (imported as SampleData[SequencesWithQuality]). To perform quality control and generate ASVs, DADA2 was used (Callahan et al., 2016) (indicating ‐‐p‐trunc‐len 290). The phylogenetic tree for the 16S rRNA amplicon set was generated with the *fragment‐insertion* plugin for SEPP using the GreenGenes database (McDonald et al., 2012), with default settings. For ITS, de novo phylogeny was created by multiple sequence alignment with mafft (Katoh & Standley, 2013). The trees were used to filter redundant features.

To perform a more accurate taxonomic classification, a pre‐fitted sklearn‐based taxonomy classifier was used with the plugin *feature‐classifier classify‐sklearn*, using default settings. The SILVA 138 (515–806—99%) and the UNITE ver8 (99%) reference data set were used to train the classifiers for 16S rRNA and ITS, respectively.

The ASVs tables were exported to R (v4.1.2), and compositional analysis was carried out using phyloseq (McMurdie & Holmes, 2013), vegan (Oksanen et al., 2013), Deseq2 (Love et al., 2014) and metacoder packages (Foster et al., 2017).

To evaluate if the diversity of the microbial samples was fully captured, a rarefaction analysis was performed using the *alpha‐rarefaction plugin*. Alpha and beta diversity analysis was conducted with the *diversity core‐metrics‐phylogenetic* plugin with a sampling depth based on the rarefaction results.

### Permutation analyses

ProgPerm (Zhang et al., 2021) was used to study the individual associations and to provide measures of the discoveries' robustness and the results' reliability. The method progressively permutes the grouping factor labels of microbiome samples and performs differential testing by applying a Kruskal–Wallis test to the permuted data in each instance. The signal strength (− log10 *p* values) of top hits from the observed data is compared with their testing performance in permuted data sets to discriminate between significant and non‐significant hits.

## RESULTS

**FIGURE 1:**
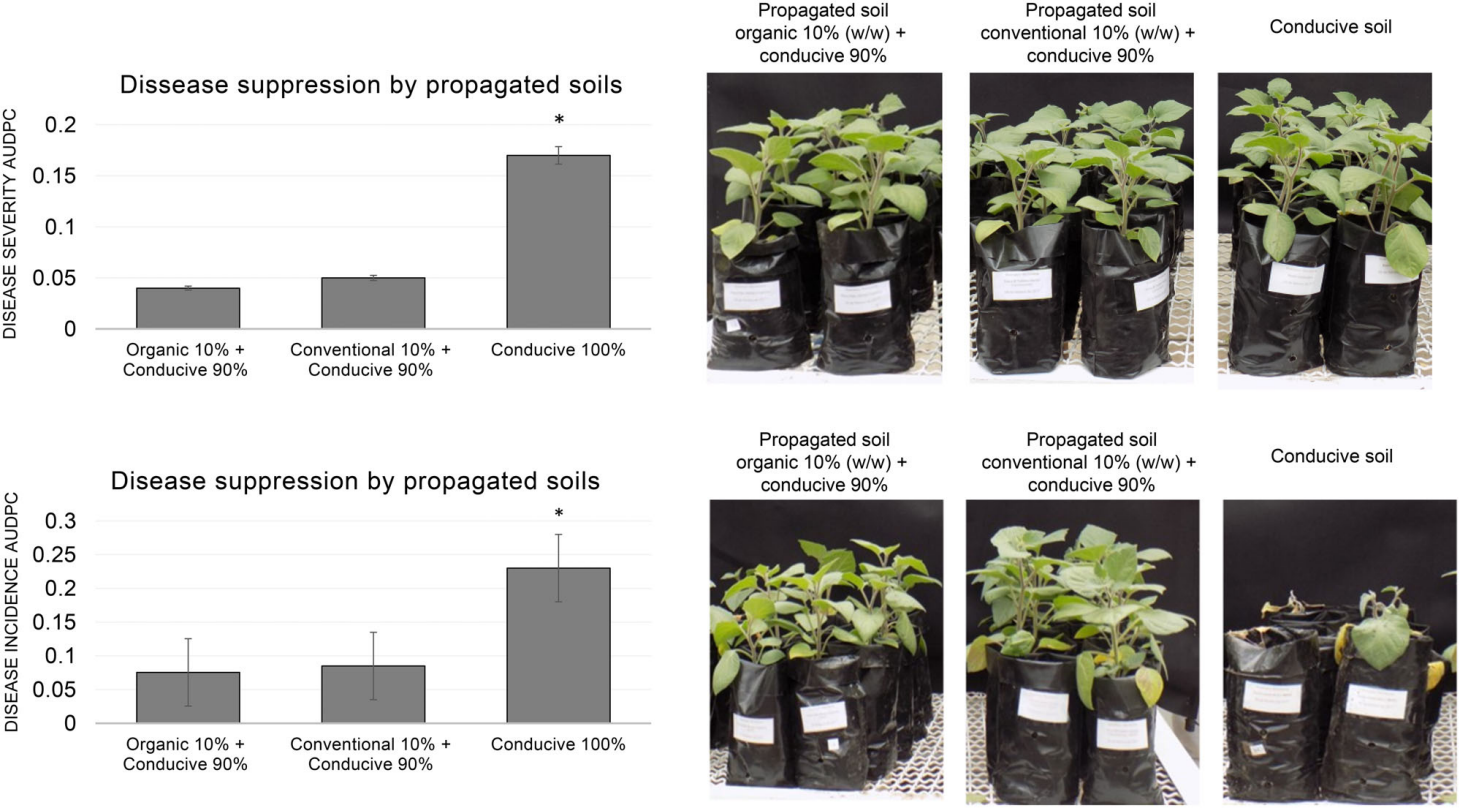
Disease severity of *Fusarium oxysporum* f sp. *physalis* (*Foph*) on propagated soils versus conducive soil. Plants sown on propagated soils (10% organic + 90% conducive soil or 10% conventional + 90% conducive soil) significantly (*p* < 0.05) reduced disease severity and disease incidence, mean area under the disease progress curve (AUDPC). The photos are from one experiment, but the results were consistent throughout all the experiments performed.

We hypothesize that soils from the Nariño department where no *F. oxysporum* f sp. *physalis* (*Foph*) has been reported on cape gooseberry plants might be suppressive against this pathogen. To test this hypothesis, we evaluated the effect of propagated soils (10% soils from two cape gooseberry farms in Nariño state and 90% of conducive soil w/w) on disease suppression of *Foph* on cape gooseberry. The two farms were selected because of their contrasting management systems: organic and conventional. Disease incidence and severity were significantly lower (*p* < 0.05) when cape gooseberry plants were grown on propagated soils (regardless of whether they came from organic or conventional farms) than when conducive soil was used (Figure 1).

To determine whether the soil suppression could be attributed to the physicochemical properties of the suppressive soils, physicochemical analysis of the suppressive, propagated, and conducive soils was done (Table 1). Except for B, P and Na, which differ greatly among all soil types, all other physicochemical properties of the propagated soils do not differ from the conducive soil used and are different from the original suppressive soils. This can be expected since propagated soils comprise 10% w/w suppressive soil and 90% w/w conducive soil. Thus, we hypothesize that the suppressive effect observed for propagated soils could be related to the microbial communities (microbiome) present in the suppressive soils rather than their physicochemical properties.

To analyse the soil microbiome from the propagated suppressive soils, libraries to identify prokaryotes (bacteria and archaea) and fungi were constructed and sequenced with MiSEQ (Illumina).

### Sequencing quality assessment

Propagated and conducive soils were sequenced based on amplicon metabarcoding. The microbial composition for the 16S rRNA and ITS rRNA samples was determined using amplicon sequence variants (ASVs). A total of 7187 and 2116 ASVs were assembled for 16S rRNA and ITS, respectively, across 80 samples.

**FIGURE 2:**
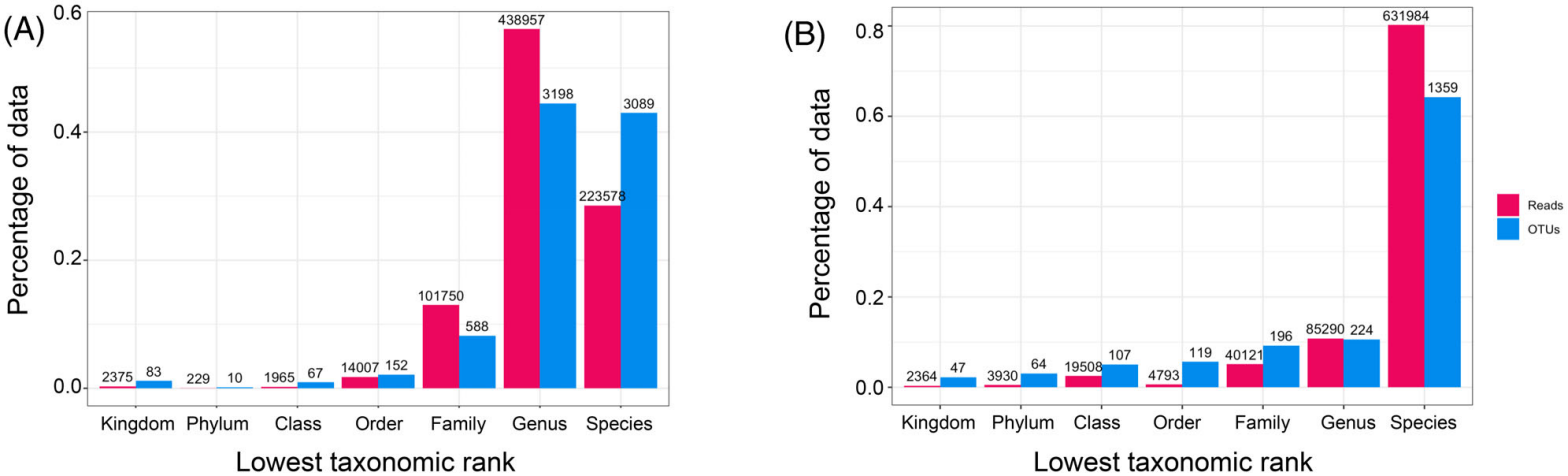
Taxonomic resolution for all samples. Percentage of data assigned to a taxonomic rank, for bacteria 16S rRNA (A) and fungi ITS reads (B). The numbers above bars indicate the ASV unique numbers for each taxonomic rank. ASV, amplicon sequence variant.

A rarefaction curve for all samples was calculated to examine whether the number of sequences obtained for each library represents the samples' diversity (Figure S1A,B). For most of the biological replicates, the rarefaction curves level off, suggesting that the present community was sufficiently characterized. Nonetheless, some technical replicates displayed a low sequencing depth (<5000 sample size) and were removed. Based on the number of replicates for each sample, there was no loss of any treatment. To uncover the proportion of reads assigned to a higher taxonomic rank, the percentage of reads and ASVs classified at their lowest taxonomic rank were plotted (Figure 2A,B). For the 16S rRNA data set, most of the reads were classified at the genus rank. Despite the number of ASVs is similar for genus and species ranks, the species reads are considerably lower than at the genus rank. This may be caused by the trained classifier's inability to assign the amplicon to a higher rank, as the quality of the reads or sequencing errors left an ambiguous match to the database. On the other hand, for ITS, there is a significant difference between the species rank and the rest. While the total number of reads and ASVs are lower than the 16S rRNA data set, the classifier assigned almost 80% of the reads to the species level. However, it must be noted that the 16S rRNA marker gene used cannot classify the species level with a high degree of certainty. A longer marker gene for ITS and 16S rRNA in the library preparation could make the assignment more specific.

### Compositional differences among soils

**FIGURE 3:**
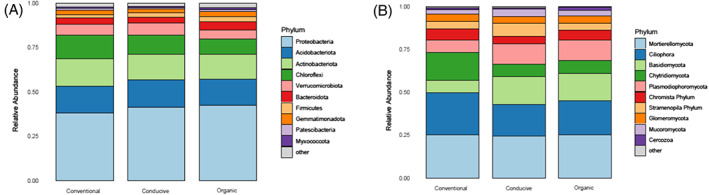
Taxonomical composition for the different types of soil analysed. (A) Based on the presence of ASVs at the Phylum rank for 16S rRNA reads and (B) ITS reads. ASV, amplicon sequence variant.

Hierarchical clustering of all samples based on their Hellinger‐transformed expression displayed no grouping at the class level for the type of soil (propagated conventional, propagated organic and conducive soil) or inoculation status (if inoculated or not with *Foph*) (Figure S2). At the ASV level, the most prevalent taxa were highly abundant across all samples, which explain the muffled clustering of biological replicates. For 16S rRNA, the top five most prevalent taxa were Alphaproteobacteria, Gammaproteobacteria, Actinobacteria, Acidobacteriae and Verrucomicrobiae (Figure S2A). For bacteria, the Phylum Proteobacteriota, Acidobacteriota, and Actinobacteriota showed the most abundant ASVs (Figure 3A). The Phylum Patescibacteria had almost two times the abundance in suppressive soils (1.27%) than in conducive soil (0.75%) (Figure 3A). For ITS, the five most prevalent taxa were the fungi Sordariomycetes, Eurotiomycetes, Mortierellomycetes, Rhizophydiomycetes and the protist Spirotrichea (Figure S2B). The most sequenced fungal species belong to the Ascomycota phylum, >87% of the sequences (Figure S3).

**FIGURE 4:**
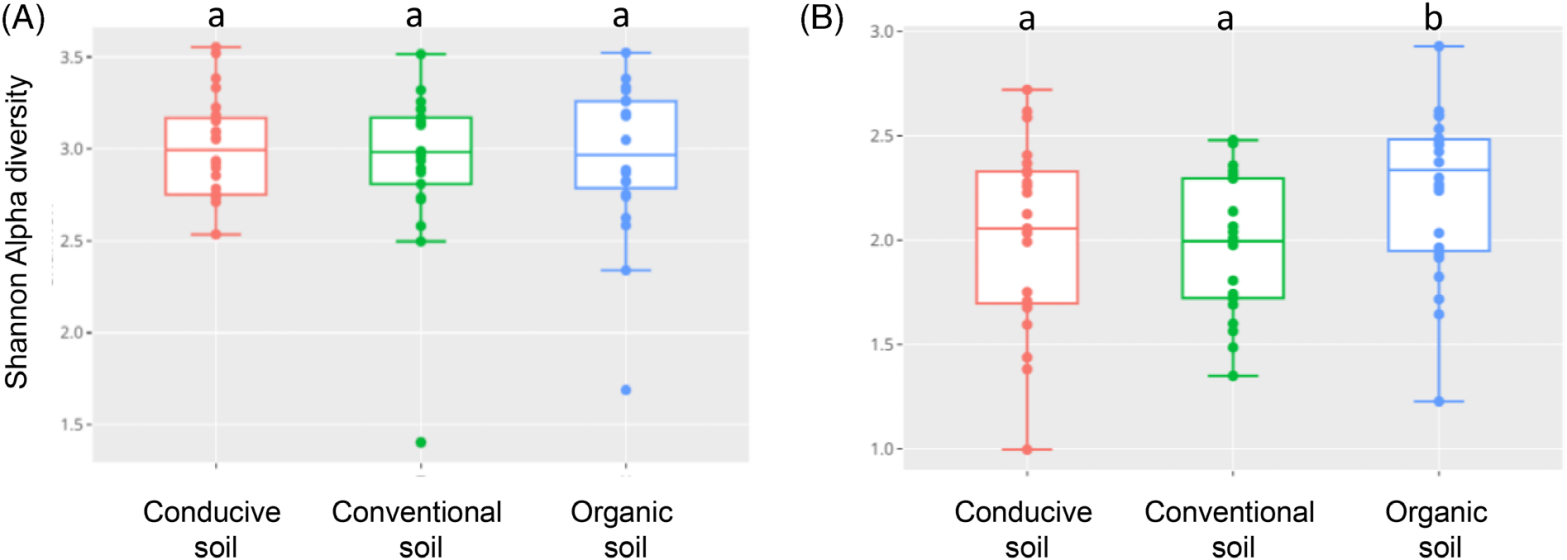
Alpha diversity analysis using the Shannon index for all samples at the Family level. Shannon index for bacteria (16S rRNA) (A) and Shannon index for fungi (ITS) (B). Shannon index shows a significant difference (*p* < 0.05, Kruskal–Wallis rank sum test) in ITS abundance for propagated organic soils.

Using the Shannon index at the Family level, alpha diversity showed significant differences (*p* < 0.05, Kruskal–Wallis rank sum test) between the different types of soils (propagated conventional, propagated organic and conducive) for the ITS amplicon set but not for the 16S rRNA (Figure 4). The propagated organic soil samples showed the highest diversity of fungi compared to the conventional soil samples, possibly due to the type of management of the cape gooseberry crop.

### Core microbiome and outliers

**FIGURE 5:**
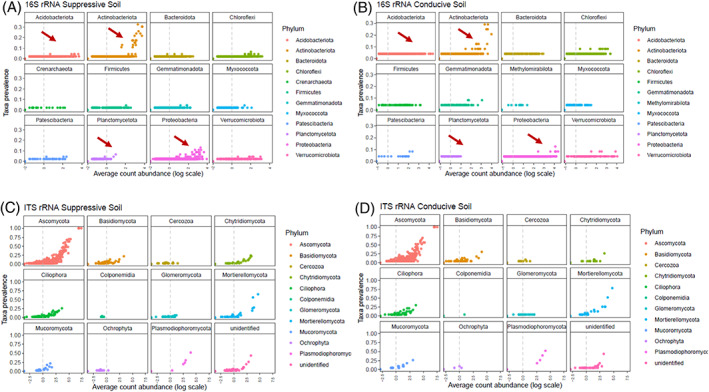
Amplicon sequence variants taxa prevalence within their Phylum. The average count abundance in the log scale, displaying the contribution of each ASV for each sample, is shown. Prevalence of the 12 top‐most abundant phyla for (A) 16S rRNA suppressive soil, (B) 16S rRNA conducive soil, (C) ITS suppressive soil and (D) ITS conducive soil. Red arrows highlight Phylum showing the difference in prevalence between suppressive and conducive soils for 16S rRNA.

To identify which ASVs belong to the core microbiome comprizing conventional and organic propagated soils, the prevalence of each ASV was plotted (Figure 5). The 12 most prevalent phyla at the Phylum level showed that most of the ASVs have low abundance. Although the taxonomic rank employed is at the Phylum level, most of the ASVs present do not represent a prevalent feature within their taxa. Thus, the presence of the relevant microorganisms in the conventional and organic soils might be attenuated by the predominant amount of conducive soil, which does not share a distinct core microbiome (Figure 5). Nonetheless, Acidobacteriota, Actinobacteriota, Planctomycetota and Proteobacteria are more prevalent in suppressive soils than in conducive soils (Figure 5A,B). However, for fungi, it appears that the prevalence of all taxa is very similar among soil samples (Figure 5C,D).

**FIGURE 6:**
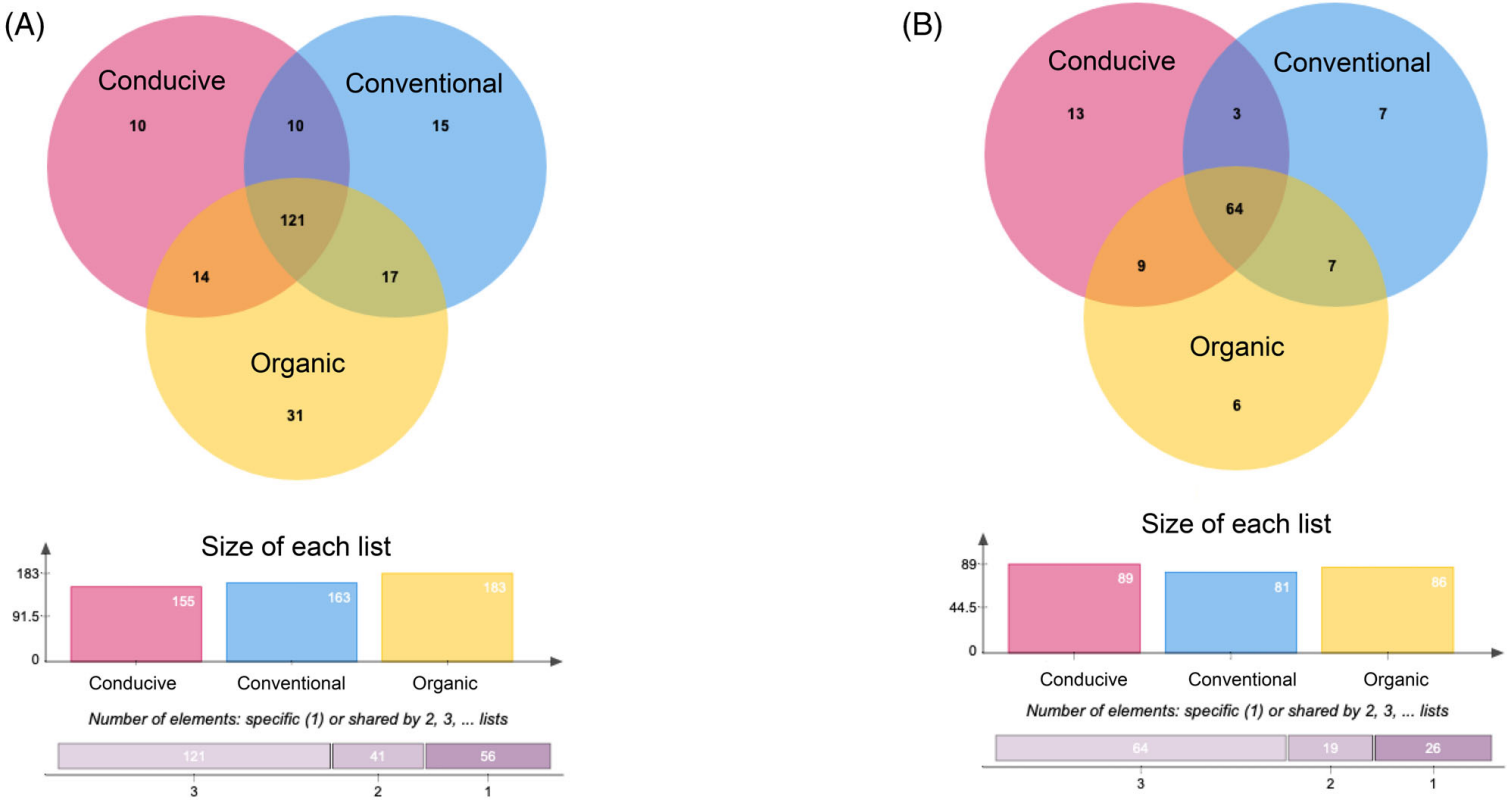
Venn diagrams show the shared and unique number of taxa for each soil type. Conducive, propagated conventional and propagated organic (A) for 16S rRNA and (B) for ITS. The list of genes for each category is available in Table S1.

Thus, the detailed search of single ASVs shared discretely between the suppressing soils seemed to be one of the options for discovering the biological mechanism of action. To this end, a Venn diagram based on ASV absolute presence on each soil type was plotted. At the Family level, propagated organic soil had more unique bacterial ASVs (32), while conducive soil had more unique fungal ASVs (13). Propagated soils shared 17 ASVs for 16S rRNA and 7 ASVs for ITS (Figure 6A,B). The complete list of genes for each category is in Table S1.

### Differentiation of microbiome compositions among soils

**FIGURE 7:**
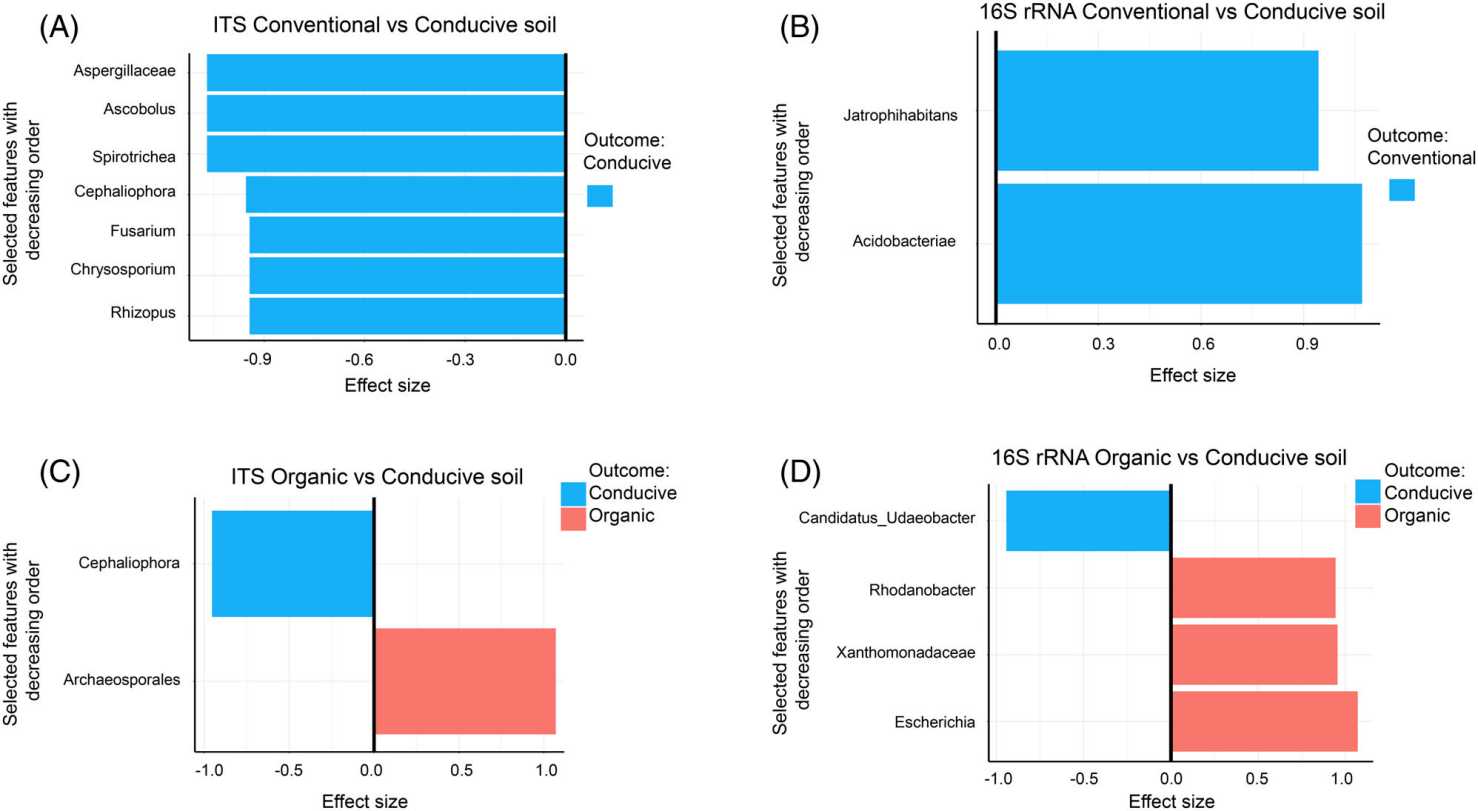
Effect size of identified features. For the taxa at the Family level that were more significantly represented between the types of soil, their effect size was estimated. Propagated conventional versus conducive soils ITS (A), 16S rRNA (B), propagated organic versus conducive soils ITS (C) and 16S rRNA (D).

Because of the high degree of heterogeneity of the soil microbiome samples, hindering the identification of differences in microbiome compositions among propagated soils and conducive soil, the ProgPerm R‐shiny tool (Zhang et al., 2021) was used. A Kruskal–Wallis test was used to compute the *p* values since it is a robust nonparametric test. Thus, to discover differences in microbiome compositions, progressive permutations of the grouping factors of the microbiome samples and multiple differential abundance tests (*p* ≤ 0.05) were performed for the taxa at the Family level. Then, the − log10 *p* values of targeted features that fell within the 95% confidence interval of the median *p* values for the complete permutation scenario are identified, and the most represented taxa for each type of soil are identified and ranked (Zhang et al., 2021). The different abundances in microbial compositions on each soil type for both bacteria (16S rRNA) and fungi (ITS) were then assessed based on their effect size. (Figure 7). For fungi abundance, the comparison between propagated conventional versus conducive soil showed that Aspergilliaceae, Ascobolus, Cephaliophora, Fusarium, Chrysosporium and Rhizopus taxa and the protist Spirotrichea were more abundant in conducive soils (Figure 7A). When comparing propagated organic soil versus conducive soil, the genus Archaeosporales was more abundant in propagated organic than conducive soil, while Cephaliophora was more abundant in conducive soil (Figure 7C). Comparing bacterial composition between propagated conventional versus conducive soil, Acidobacteria and Jatrophohabitans were more abundant for propagated conventional soil (Figure 7B), while for propagated organic vs conducive soil, the most abundant bacteria were Escherichia, Shigella, Xanthomonadaceae and Rhodanobacter, while for conducive soils was Candidatus Udaeobacter (Figure 7D).

## DISCUSSION

Fusarium wilt is an important disease in a vast number of economically important crops worldwide, such as tomato (Prihatna et al., 2018), banana (Pegg et al., 2019) and cape gooseberry (Simbaqueba et al., 2018). It is a soil‐borne pathogen that is extremely difficult to manage because it can remain in the soil for long periods even when the host crop is absent (Pegg et al., 2019). Suppressive soils protect plants against soil‐borne pathogens in a microbiome‐mediated manner (Gómez Expósito et al., 2017). The present work studied microbial communities of Fusarium wilt suppressive and conducive soils. As the experimental propagated soils share 90% of conducive soil, it was expected to see a similar taxa composition among all soil types. Clear discrimination of bacterial and fungal communities based on soil types was not observed. Nevertheless, both propagated soils were suppressive against *Foph*, suggesting that the taxa that may induce such properties were still present, and represented low prevalence and abundance. For instance, propagated organic and conventional soils presented the phylum Planctomycetota, which has been associated with suppressive soils, in higher prevalence and abundance than conducive soil (Chapelle et al., 2015; Köberl et al., 2020; Sanguin et al., 2009). However, there was no difference in bacterial abundance (Shannon index) among soils. These results suggest that the relative abundance of some bacterial taxa might be a more important indicator of disease suppression than the exclusive presence of specific taxa. For example, the Phyla Acidobacteriota, Actinobacteriota and Proteobacteria are present both in conducive and suppressive soils, but the ASVs for these taxa seem to be more prevalent in suppressive soils (Figure 7A,B), as reported previously (Gómez Expósito et al., 2017; Köberl et al., 2020; Siegel‐Hertz et al., 2018; Toyota & Shirai, 2018; Xiong et al., 2017).

The taxonomical composition at the Phylum rank for fungi was similar among the different soils. However, the Shannon index shows a significant difference (*p* < 0.05) in ITS abundance for propagated organic soils, which could be a consequence of the organic management versus the conventional management with pesticide use that may reduce fungal diversity. Fungal abundance comparison between propagated conventional vs conducive soil showed that Aspergilliaceae, Ascobolus, Cephaliophora, Fusarium, Chrysosporium and Rhizopus taxa together with the protist Spirotrichea, were more abundant in conducive soils. These results are interesting since some of these taxa are plant pathogens such as Fusarium, Cephaliophora and Rhizopus, and some species are enriched in conducive soils (Petrasch et al., 2019; Sweta et al., 2014; Wang et al., 2020; Xiong et al., 2017).

Studies of soil microbiomes have uncovered the complex mechanisms governing suppressive soils and have shown that the complexity of soil microbiomes can be overwhelming. The advent of high‐throughput sequencing has permitted a holistic study of soil microbiomes in a vast number of pathosystems and geographic distributions (Döring et al., 2020; Gómez Expósito et al., 2017; Köberl et al., 2020; Petrasch et al., 2019; Siegel‐Hertz et al., 2018; Wang et al., 2020; Xiong et al., 2017). These studies show that despite the high diversity of soil microbiomes, the consistent presence of some bacterial and fungal taxa in suppressive soils might indicate that soil microbiome composition is a crucial factor for plant health, suggesting that despite the complexity of microbial communities, some predictive microbial diversity models can be used as biomarkers for healthy plant microbiomes for biocontrol strategies. The next step will be to challenge this hypothesis by selecting some of the microbial markers to assess for plant health both under control conditions and in the field.

## AUTHOR CONTRIBUTIONS

**Daniel Bautista:** Data curation (lead); formal analysis (equal); methodology (lead); writing – original draft (equal). **Diana García:** Investigation (equal); methodology (equal); writing – review and editing (supporting). **Lorena Dávila:** Investigation (equal); methodology (equal); writing – review and editing (supporting). **Alejandro Caro‐Quintero:** Data curation (supporting); formal analysis (supporting); investigation (supporting); methodology (supporting); writing – original draft (supporting). **Alba Marina Cotes:** Conceptualization (lead); formal analysis (equal); funding acquisition (lead); methodology (equal); project administration (lead); writing – review and editing (supporting). **Adriana González:** Investigation (supporting); methodology (supporting); supervision (supporting); writing – review and editing (supporting). **Paola Zuluaga:** Data curation (equal); formal analysis (lead); investigation (lead); methodology (equal); writing – original draft (lead).

## CONFLICT OF INTEREST STATEMENT

The authors declare no conflicts of interest.

## Supporting information


**FIGURE S1.** Rarefaction curves. Evaluating the species richness based on depth of sequencing for bacteria 16S rRNA (a) and fungi ITS (b) amplicon sets for conducive soil and propagated conventional and organic soils.

**FIGURE S2.** Hierarchical clustering of all samples based on their Hellinger‐transformed expression. No grouping at class level for type of soil (conventional, organic and conducive soil) or inoculated status (if inoculated or not with Foph) for either bacteria (A) or fungi (B).

**FIGURE S3.** Taxonomical composition for the different types of soil analysed. ITS reads highlighting that, on average, >90% of the sequences are Ascomycota (conventional 93.3%, conducive 90.3% and organic 87.2%).

**TABLE S1.** Venn diagrams for conducive, propagated conventional and propagated organic for ITS.

## Data Availability

The data sets generated during and analysed during the current study are available from the corresponding author upon reasonable request.
