# Supplementary material for: Studying the microbiome of suppressive soils against vascular wilt, caused by Fusarium oxysporum in cape gooseberry (Physalis peruviana)
Source: Environ Microbiol Rep. 2023 Sep 7;15(6):757–68. doi: 10.1111/1758-2229.13195 (PMC10667652; doi:10.1111/1758-2229.13195)
Supplement: Supplementary file 1 — FIGURE S1. Rarefaction curves. Evaluating the species richness based on depth of sequencing for bacteria 16S rRNA (a) and fungi ITS (b) amplicon sets for conducive soil and propagated conventional and organic soils. [file EMI4-15-757-s002.pptx]

## Slide 1
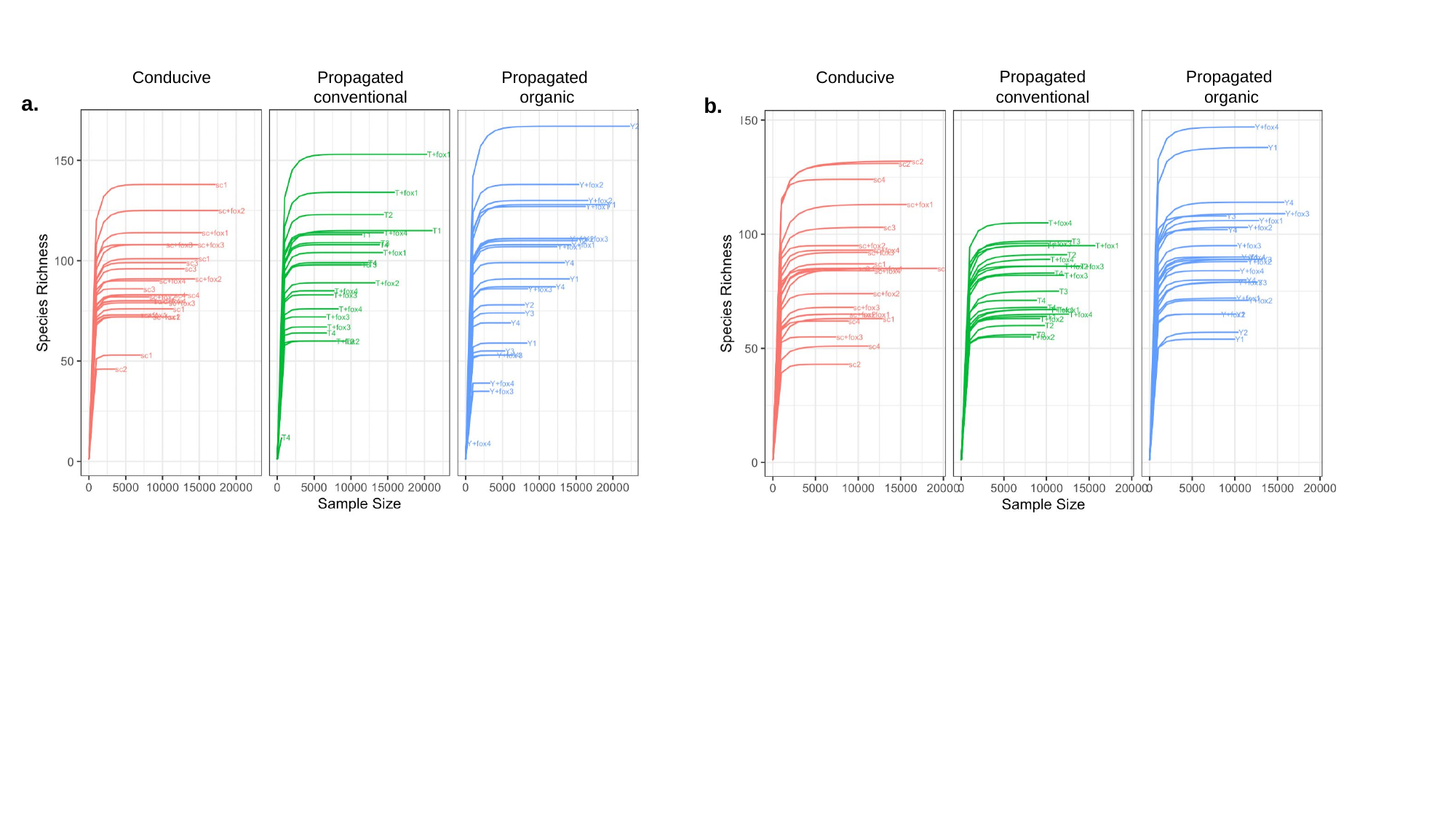

a.
Propagated
organic
b.
Propagated conventional
Conducive
Propagated
organic
Conducive
Propagated conventional
