# Supplementary figures and images for: Studying the microbiome of suppressive soils against vascular wilt, caused by Fusarium oxysporum in cape gooseberry (Physalis peruviana)
Source: Environ Microbiol Rep. 2023 Sep 7;15(6):757–68. doi: 10.1111/1758-2229.13195 (PMC10667652; doi:10.1111/1758-2229.13195)

## Slide 1
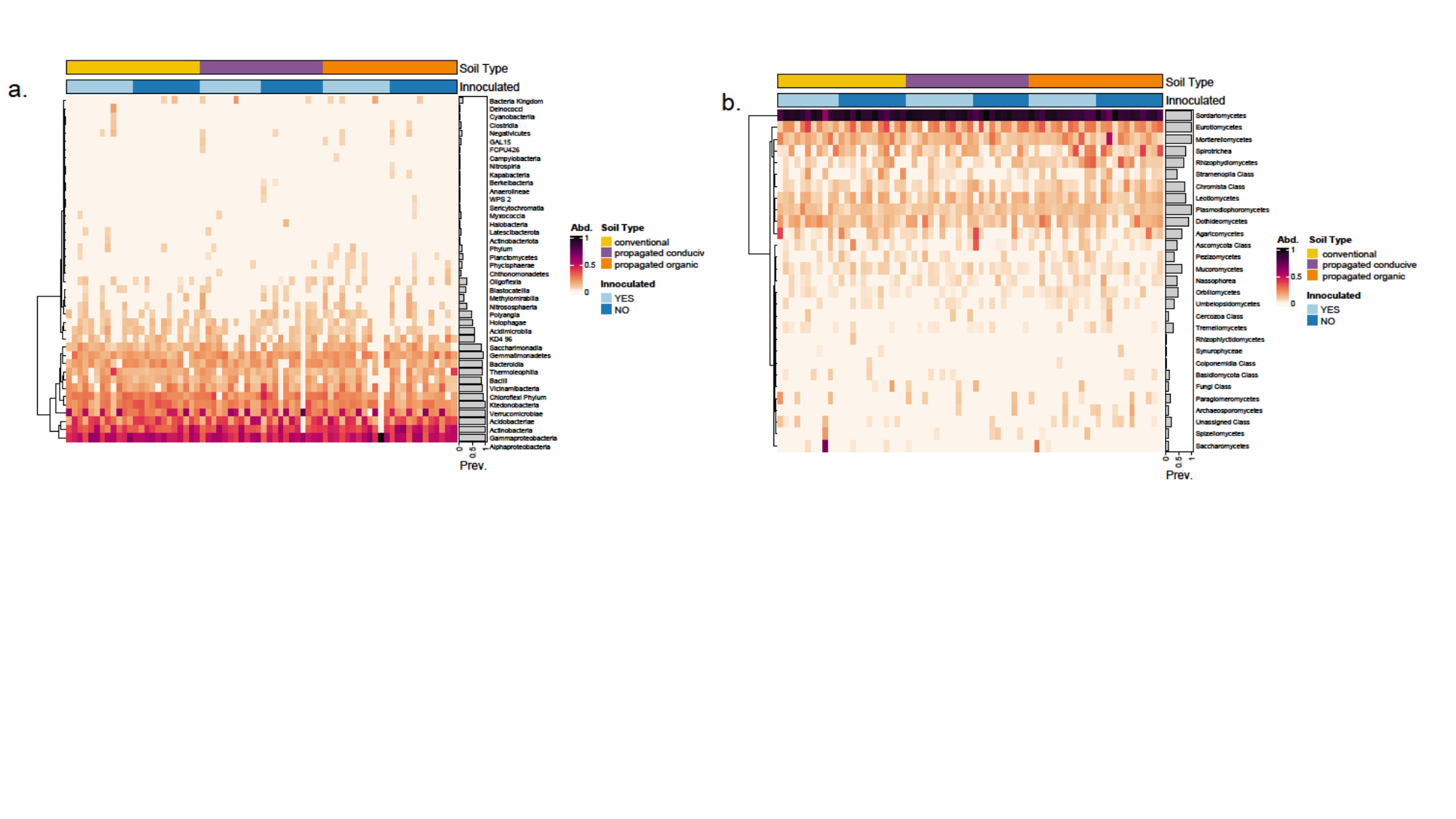

#

Supplement: Supplementary file 2 — FIGURE S2. Hierarchical clustering of all samples based on their Hellinger‐transformed expression. No grouping at class level for type of soil (conventional, organic and conducive soil) or inoculated status (if inoculated or not with Foph) for either bacteria (A) or fungi (B). [file EMI4-15-757-s001.pptx]

## Slide 1
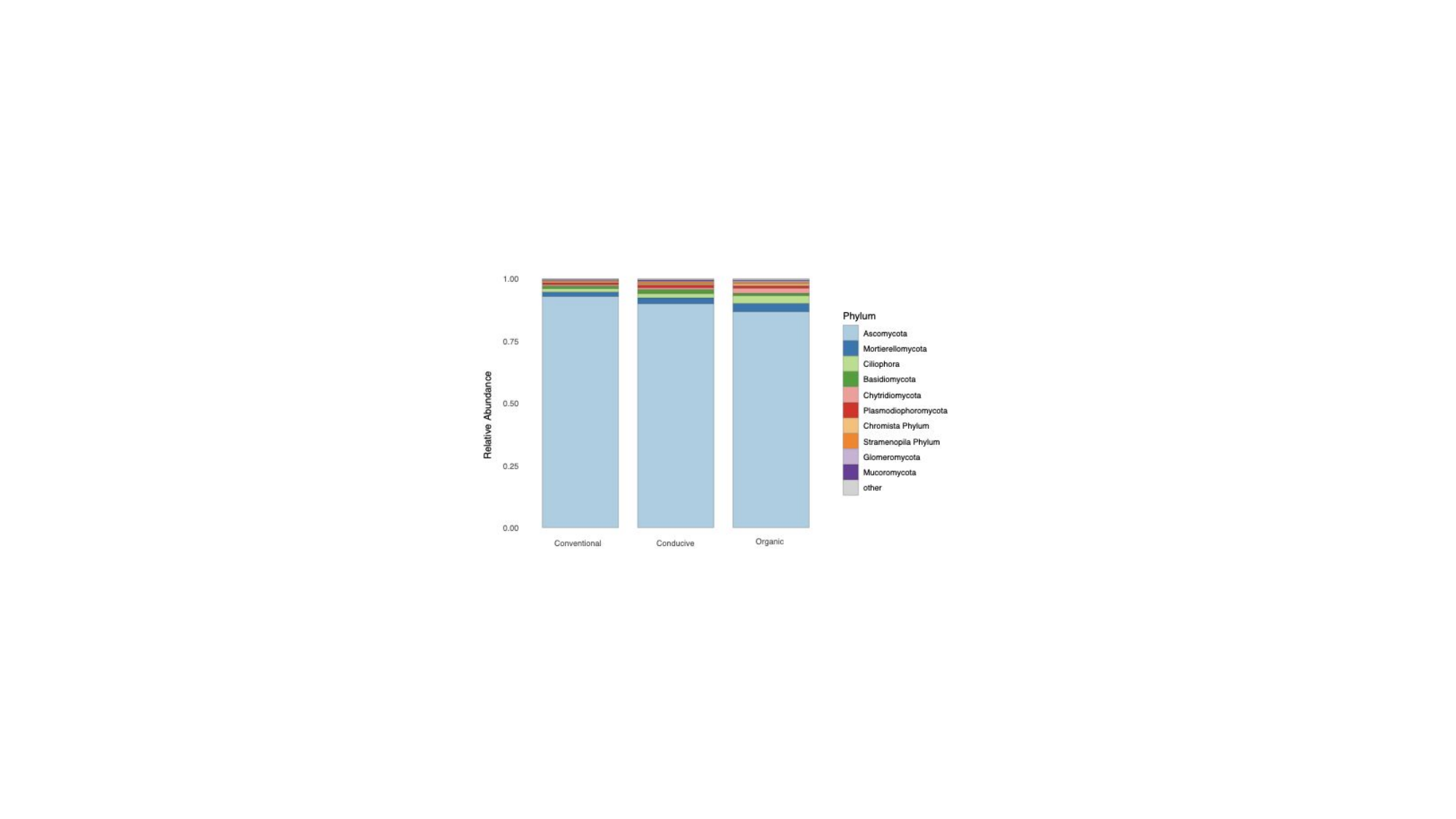

#

Supplement: Supplementary file 3 — FIGURE S3. Taxonomical composition for the different types of soil analysed. ITS reads highlighting that, on average, >90% of the sequences are Ascomycota (conventional 93.3%, conducive 90.3% and organic 87.2%). [file EMI4-15-757-s004.pptx]
